# Cutaneous immune-related adverse events among Taiwanese cancer patients receiving immune checkpoint inhibitors link to a survival benefit

**DOI:** 10.1038/s41598-022-11128-5

**Published:** 2022-04-29

**Authors:** Yung-Tsu Cho, Yi-Tsz Lin, Che-Wen Yang, Chia-Yu Chu

**Affiliations:** 1 Department of Dermatology, National Taiwan University Hospital, National Taiwan University College of Medicine, 15F, No.7 Chung-Shan South Road, Taipei, 10002 Taiwan; 2 Department of Dermatology, Fu Jen Catholic University Hospital, Fu Jen Catholic University, New Taipei City, Taiwan; 3 Department of Dermatology, Wan Fang Hospital, Taipei Medical University, Taipei, Taiwan; 4 Department of Dermatology, Cathay General Hospital, Taipei, Taiwan; 5 School of Medicine, College of Medicine, Fu Jen Catholic University, New Taipei City, 24205 Taiwan

**Keywords:** Medical research, Oncology, Signs and symptoms, Immunological disorders, Immunotherapy, Skin diseases

## Abstract

Cutaneous immune-related adverse events are common in cancer patients receiving immunotherapies but seldom studied in a comprehensive way of collecting all cancer types with comparisons between different immune-oncology drugs and correlation to patient survival. In this retrospective cohort study, we recruited 468 cancer patients receiving immunotherapies in a tertiary referral center in Taiwan and try to determine real-world incidence of cutaneous immune-related adverse events and their associations with the survival rates. Among them, 128 patients (27.4%) had cutaneous immune-related adverse events, with maculopapular eruption (10.6%) and pruritus (10.1%) most frequently identified in the monotherapy group. The incidence of these cutaneous immune-related adverse events was highest in patients receiving pembrolizumab (34.1%, *P* < .0001). Concurrent usage of molecular-targeted therapy with immunotherapy was associated with a higher incidence (57.8%, *P* < .0001). The Kaplan–Meier plot and log-rank test showed that patients with any type of immune-related cutaneous adverse events had longer survival time than those without (*P* < .0001). In conclusion, having either type of cutaneous immune-related adverse event in cancer patients receiving immunotherapies was correlated with a longer overall survival. Prompt diagnosis and suitable treatment are important.

## Introduction

Immune checkpoint inhibitors (ICIs), including anti-programmed death-1 (anti-PD-1), anti-programmed death ligand-1 (anti-PD-L1), and anti-cytotoxic T-cell lymphocyte-associated antigen-4 (anti-CTLA-4) monoclonal antibodies, represent a breakthrough in cancer therapy and have shown great benefits in the treatment of multiple cancer types, besides melanoma, and especially in non-small cell lung cancer (NSCLC) and head and neck squamous cell carcinoma (HNSCC)^1^. Despite their benefit, immune-related adverse events (irAEs) potentially involving multiple organ systems may cause treatment discontinuation in severe conditions. Cutaneous immune-related adverse events (cutaneous irAEs) are the most frequently encountered ones in clinical practice, and usually the first to appear with a wide range of manifestations^1^. Time to onset of cutaneous irAEs ranges from weeks to months, but delayed onset after the termination of medication has been seen as well^1,2^.

Various types s of cutaneous irAEs including maculopapular eruption (MPE), pruritus, and eczematous eruption have been described^3–5^. Rare manifestations, like bullous pemphigoid (BP), psoriasiform eruption, and vitiligo, have been reported to be associated with the clinical benefit of tumor response; or the life-threatening Stevens-Johnson syndrome/toxic epidermal necrolysis (SJS/TEN) warrants medication discontinuation^3,5–8^. However, cutaneous irAEs have been mostly studied in clinical trials, Caucasians, or melanoma patients using general terms without being systemically delineated in dermatologic terms^9^.

Given the critical need to gain familiarity with the cutaneous irAEs in both oncologists and dermatologists, our study aims to provide real-world incidence, manifestations, and illustration with the correlation of patient survival with cutaneous irAEs from a tertiary referral center with oncology department, dermatology department, and an integrated clinic of both specialties.

## Results

### Demographics of the patients

**Figure 1:**
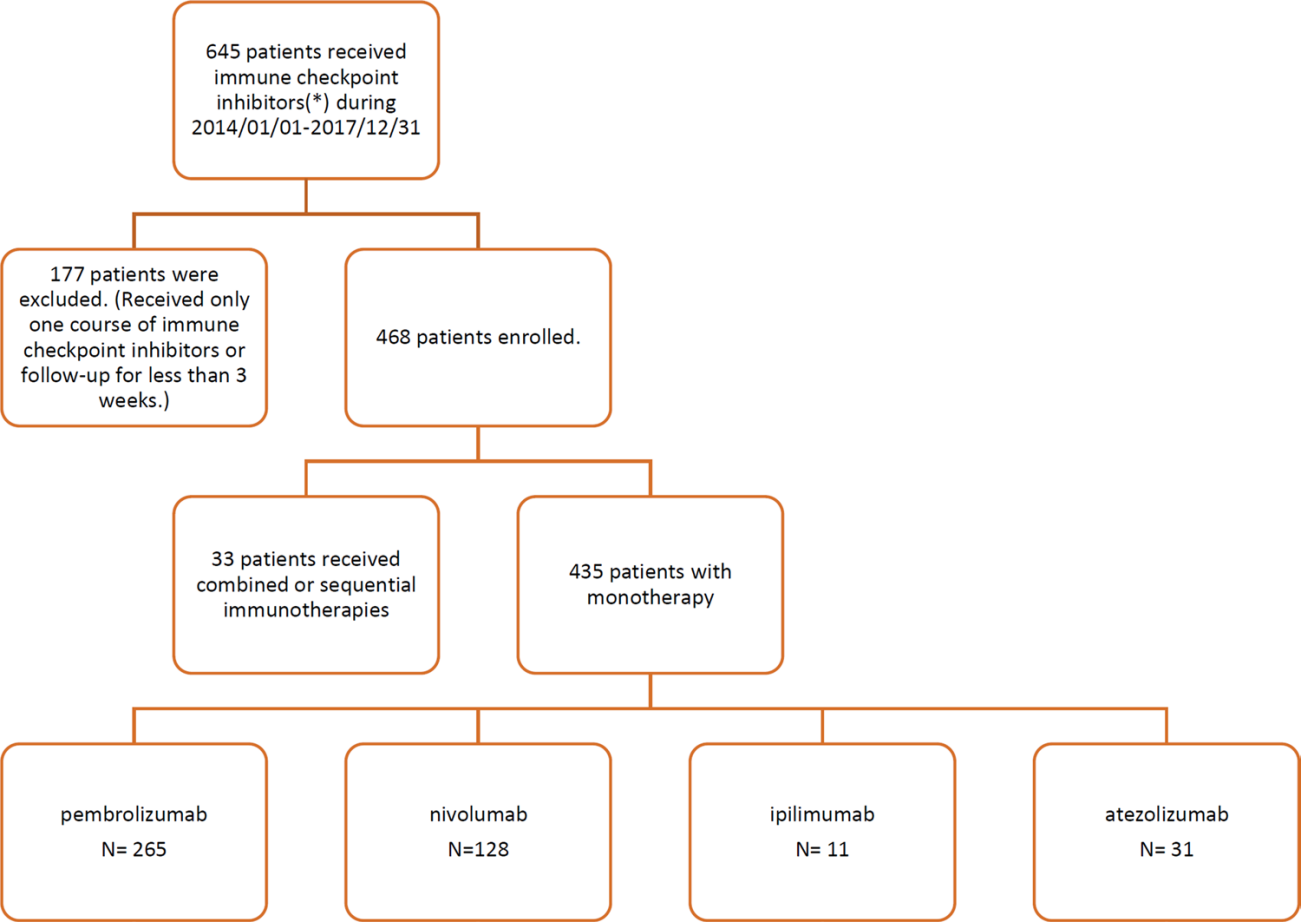
Study protocol and patient enrollment. Immune checkpoint inhibitors: pembrolizumab, nivolumab, ipilimumab, atezolizumab.

After excluding patients who did not fulfill the inclusion criteria, the records of 468 eligible cancer patients were retrieved, and 435 of them had received monotherapy, including 265 using pembrolizumab, 128 receiving nivolumab, 11 receiving ipilimumab, and 31 using atezolizumab, and more than one agent were used either in combination or sequentially in 33 patients (Fig. 1). The median follow-up duration of these patients were 205 days (interquartile range 90–370 days). Among the 468 patients, 128 were diagnosed as having cutaneous irAEs, and the overall incidence was 27.4% (0.37 per person-year). The median follow-up durations for patients with and without cutaneous irAEs were 302 days (interquartile range 190.5–451 days) and 159.5 days (interquartile range 75–331.3 days), respectively. Thirteen (10.2%) of the patients with cutaneous irAEs had received skin biopsies and revealed 3 SJS/TEN, 2 lichenoid eruptions, 2 eczematous, 2 psoriasis, and 1 BP. The incidences were similar between monotherapy and combination or sequential therapies of the immune checkpoint inhibitors (Table 1). Considering the underlying cancer types, the included patients were mainly diagnosed as having NSCLC, HNSCC, and hepatocellular carcinoma (HCC), followed by melanoma and urothelial carcinoma. The incidence of cutaneous irAEs is highest among the HNSCC group (54.4%), followed by small-cell lung cancer, nasopharyngeal carcinoma, NSCLC, urothelial carcinoma, esophageal cancer, melanoma, etc.

**Table 1:** Demographics and incidence of cutaneous irAEs.

	Enrolled patients	Patients with cutaneous irAEs		Patients without cutaneous irAEs	
	Number	Number	Percentage	Number	Percentage
Total	468	128	27.4%	340	72.6%
Monotherapy	435	118	27.1%	317	72.9%
Combination/Sequential therapy	33	10	30.3%	23	69.7%
Age (Average ± SD)	59.6 ± 13.3	61.4 ± 11.7	59.0 ± 13.8		
**Gender**					
Female	162	36	22.2%	126	77.8%
Male	306	92	30.1%	214	69.9%
**Cancer types**					
Melanoma	26	7	26.9%	19	73.1%
Non-small cell lung cancer	123	37	30.1%	86	69.9%
Small cell lung cancer	4	2	50.0%	2	50.0%
Head and neck squamous cell carcinoma	79	43	54.4%	36	45.6%
Hepatocellular carcinoma	66	5	7.6%	61	92.4%
Cholangiocarcinoma	12	1	8.3%	11	91.7%
Urothelial carcinoma	25	7	28.0%	18	72.0%
Renal cell carcinoma	6	0	0.0%	6	100.0%
Nasopharyngeal carcinoma	16	6	37.5%	10	62.5%
Breast cancer	14	2	14.3%	12	85.7%
Colon cancer	11	1	9.1%	10	90.9%
Esophageal cancer	11	3	27.3%	8	72.7%
Gastric adenocarcinoma	14	1	7.1%	13	92.9%
Pancreatic cancer	6	1	16.7%	5	83.3%
Hodgkin lymphoma	3	0	0.0%	3	100.0%
Others^a^	50	12	24.0%	38	76.0%
Double cancer	2	0	0.0%	2	100.0%

Diffuse intrinsic pontine glioma, metastatic cancer, precursor T-cell lymphoblastic lymphoma, anaplastic oligodendroglioma, endometrioid adenocarcinoma, glioblastoma, epithelioid sarcoma, acute myeloid leukemia, salivary adenocarcinoma, osteosarcoma, ear adenoid cystic carcinoma, Merkel cell carcinoma, parotid adenocarcinoma, uterine sarcoma, ovarian cancer, thymic cancer, diffuse large B cell lymphoma, thyroid cancer, sarcomatoid carcinoma, chondrosarcoma, pleomorphic sarcoma, mesothelioma, palate spindle cell carcinoma, lymphoepithelioma-like carcinoma, adult T-Cell leukemia/lymphoma, pre B-cell lymphoblastic leukemia, leiomyosarcoma.

*irAEs* immune-related adverse events.

^a^Others.

### Patients who received pembrolizumab had the highest incidence to develop cutaneous irAEs among all the patients in the monotherapy group

**Figure 2:**
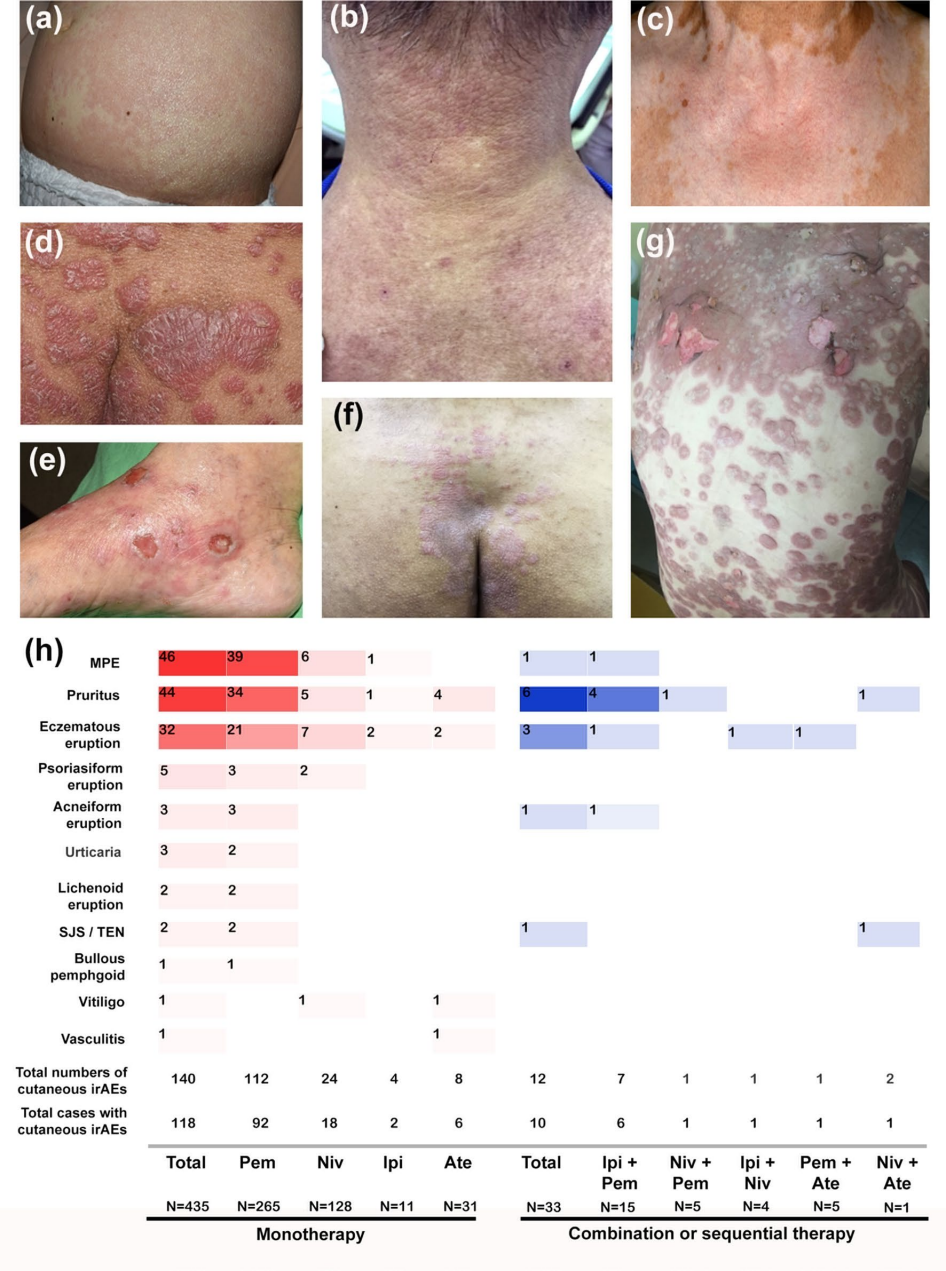
The subtypes of cutaneous irAEs in patients receiving ICIs. (**a**) MPE, (**b**) eczematous eruption, (**c**) vitiligo, (**d**) psoriasiform eruption, (**e**) bullous pemphigoid, (**f**) lichenoid eruption, (**g**) SJS/TEN and (**h**) the distribution of the subtypes of cutaneous irAEs in patients receiving monotherapy or combination/sequential therapy.

The incidence of cutaneous irAEs was analyzed based on the diagnosis and inhibitor used (Fig. 2). MPE was the most prevalent cutaneous irAE, affecting 10.6% of participants receiving the monotherapy (n = 435), followed by pruritus and eczematous eruption (10.1% and 7.4%). Some common cutaneous irAEs may co-exist, such as pruritus and MPE (n = 7), pruritus and eczema (n = 5), MPE and eczema (n = 3), pruritus and urticaria (n = 3), and others. Less common cutaneous irAEs, including psoriasiform eruption, lichenoid eruption, along with the relatively rare BP and SJS/TEN, were seen and confirmed by histopathology examination. Vitiligo, which was common in the melanoma group, was found in only one patient. The development of cutaneous irAEs was significantly more frequent in the pembrolizumab group (34.7%) than in the atezolizumab group (19.4%), the ipilimumab group (18.2%), and the nivolumab group (14.1%) (*P* < 0.0001) (Fig. 2). The overall incidence of cutaneous irAEs in patients receiving the combination or sequential therapy (n = 33) were similar to those receiving the monotherapy (30.3% and 27.1%, respectively).

### Concomitant use of molecular targeted therapy and immunotherapy harbors the highest incidence to have cutaneous irAEs

**Figure 3:**
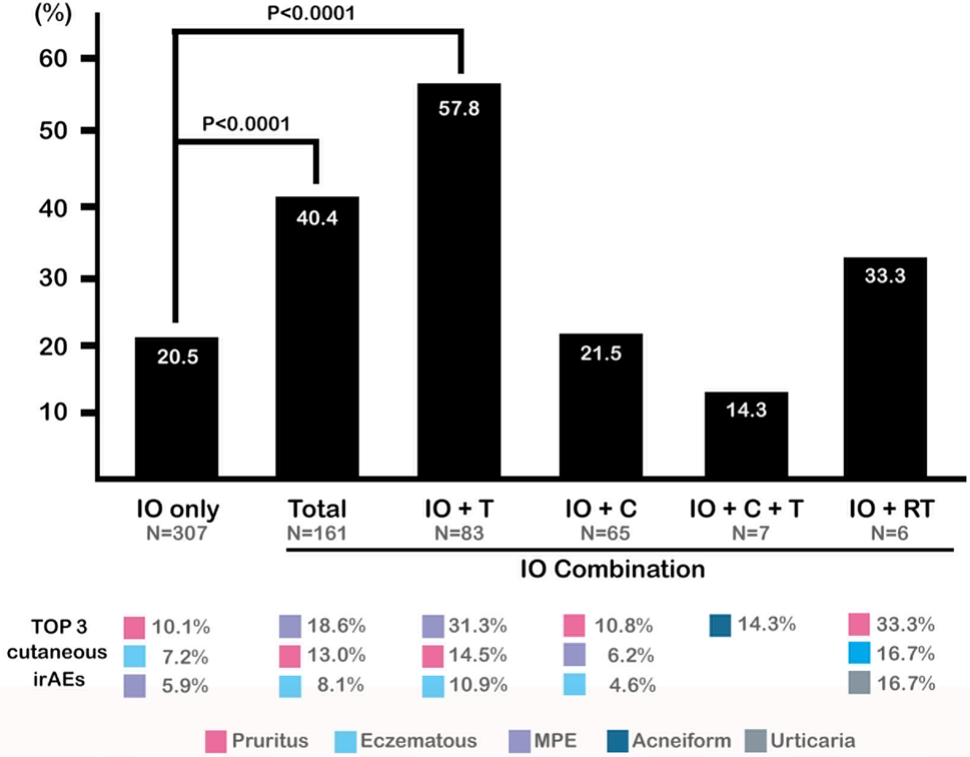
Patients receiving concomitant oncological treatments had a higher incidence of cutaneous irAEs. *C* chemotherapy; *IO* immune-oncological treatment; *irAEs* immune-related adverse events; *T* molecular target therapy; *RT* radiotherapy.

Analysis of concomitant oncological treatments (Fig. 3) showed a significant different incidence of cutaneous irAEs between patients with immunotherapy alone (63/307, 20.5%) and those with concomitant treatments (65/161, 40.4%) (*P* < 0.0001). Subgroup analysis using chi-square test and application of Bonferroni test revealed that concomitant molecular targeted therapy with immunotherapy has a highest incidence of cutaneous irAEs (48/83, 57.8%, *P* < 0.0001). Of note, MPE is the most common cutaneous irAE among patients of this subgroup, while pruritus is the most common one among patients of other subgroups.

Since the frequency of concomitant use of oncological treatments may differ among patients receiving different immune checkpoint inhibitors, we used multivariable logistic regression analysis to evaluate the impacts of each factor. After analyzing, in patients of monotherapy group, both use of pembrolizumab and concomitant use of molecular targeted therapy still showed a significantly higher risk to develop cutaneous irAEs (*P* = 0.003 and *P* < 0.001, respectively). On the other hand, use of nivolumab, atezolizumab, and ipilimumab did not show significantly increased risk of development of cutaneous irAEs.

### Patients who developed cutaneous irAEs showed better survival benefits than those who had no cutaneous irAEs

**Figure 4:**
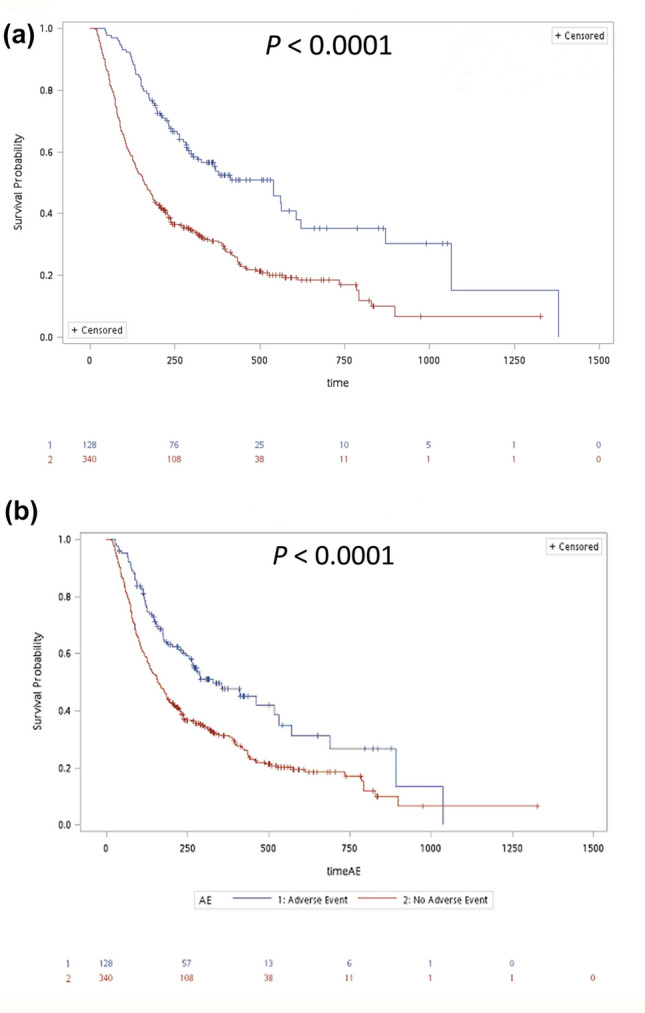
Patients with cutaneous irAEs had more survival benefits than those without cutaneous irAEs. (**a**) The Kaplan–Meier plot and log-rank test demonstrated that patients who suffered from cutaneous irAEs lived longer than those who did not. (**b**) Using the conditional landmark analysis, patients with cutaneous irAEs showed a longer survival time than those without cutaneous irAEs.

The Kaplan–Meier plot and log-rank test demonstrated that patients who suffered from cutaneous irAEs lived longer than those who did not (mean values 604.0 ± 62.6 days vs. 297.5 ± 17.4 days, *P* < 0.0001) (Fig. 4a), and subgroup analysis echoed that the survival time of patients without cutaneous irAEs was shorter than any subgroup of patients with different cutaneous irAEs (*P* < 0.0001), while no superiority of any type was established after application of the Bonferroni correction (Supplementary Fig. 1). In subsequent conditional landmark analysis to minimize the guarantee-time bias, patients with cutaneous irAEs still showed longer survival time than those without cutaneous irAEs (mean values 454.5 ± 44.1 days vs. 297.5 ± 17.4 days, *P* < 0.0001) (Fig. 4b). Similar results were also obtained in subgroup analysis (Supplementary Fig. 2, *P* = 0.0032). For further investigating the influence of cutaneous irAEs on patients’ survival, we also used a Cox regression model with consideration of age, sex, treatments, and cancer types, and found that patients who suffered from cutaneous irAEs was still a significant factor that contribute to patient survival (Table 2).

**Table 2:** Cox regression model to evaluate the influencing factors on survival of these patients.

Parameter	Univariable Cox				Multivariable Cox			
	HR	95% HR CI		*P* value	HR	95% HR CI		*P* value
Therapy: Combination/Sequential therapy	0.805	0.531	1.220	0.3061	0.817	0.526	1.269	0.3686
Age	0.992	0.984	1.000	0.0510	0.992	0.983	1.001	0.0691
Gender: Male	1.041	0.826	1.311	0.7343				
**Cancer type (Ref: Melanoma)**								
HCC	**2.274**	**1.349**	**3.834**	**0.0020**	**1.896**	**1.095**	**3.285**	**0.0224**
HNSCC	0.763	0.444	1.309	0.3257	0.709	0.401	1.256	0.2389
NSCLC	0.753	0.453	1.25	0.2725	0.662	0.387	1.130	0.1305
Others	1.225	0.753	1.992	0.4132	1.001	0.595	1.685	0.9956
UC	0.934	0.484	1.803	0.8393	0.872	0.446	1.707	0.6905
Cutaneous irAEs	**0.580**	**0.443**	**0.761**	** < .0001**	**0.687**	**0.519**	**0.910**	**0.0090**

Others included all the cancer types listed in the Table 1 other than melanoma, hepatocellular carcinoma, head and neck squamous cell carcinoma, non-small cell lung cancer, and urothelial carcinoma.

*CI* confidence interval, *HCC* hepatocellular carcinoma, *HNSCC* head and neck squamous cell carcinoma, *HR* harzard ratio, *irAEs* immune-related adverse events, *NSCLC* non-small cell lung cancer, *UC* urothelial carcinoma.

Significant values are in bold.

## Discussion

Dermatologic toxicities are the most prevalent irAEs with the overall incidences of all-grade dermatologic toxicities in patients treated for advance melanoma in clinical trials were 34–42% in nivolumab, 43.5–58.5% in ipilimumab, and 43.2–46.3% in pembrolizumab, with a higher incidence (58.5–71.5%) in patients treated with a combination of nivolumab and ipilimumab^8–10^. The integrated incidence of pruritus and rash in clinical trials was also highest among the combined administration of nivolumab and ipilimumab^9^. Among non-Asian patients receiving combination therapy, MPE was the predominant cutaneous irAE^11^. In contrary, no significant difference in the incidence of cutaneous irAEs between the monotherapy and combination therapy groups was found in our study, possibly limited by the small case number in the combination group. On the other hand, in previous reports, the incidence rate of cutaneous irAEs was usually higher in patients receiving anti-CTLA-4 drugs than those receiving anti-PD-1/PD-L1 drugs^4,8,12^. However, it is different from our findings and it is possibly resulted from the fact that prior studies did not appropriately consider malignancy type as a confounder (melanoma is an independent risk factor for cutaneous toxicities), overestimating the rate of cutaneous irAEs in ipilimumab monotherapy^13^. In addition, though the overall incidence of cutaneous irAEs in our study was relatively lower than that reported before, it was comparable to one US-based population study which showed the overall incidence of 25.1%^13^. Another interesting finding of our study is that male patients significantly outnumber than female patients. It might be due to the nature that higher incidence rates of NSCLC, HNSCC, and HCC in male patients in Taiwan^14^.

When analyzed based on underlying cancer, the highest frequency of skin adverse events reported in the literature were usually among melanoma, NSCLC, and renal cell carcinoma^3,13,14^. In our study, the incidence of cutaneous irAEs was highest among HNSCC (54.4%), while approximately 20–30% of patients treated presented with cutaneous irAEs in the major cancer groups in decreasing trend, including NSCLC, urothelial carcinoma, and melanoma, followed by 7.6% in HCC. The surprisingly high percentage of cutaneous irAEs in the HNSCC group is possibly related to the frequently concomitantly used afatinib in the treatment protocol when treating HNSCC, since afatinib and other members of epidermal growth factor receptor tyrosine kinase inhibitors may induce keratinocyte injury, and might contribute to skin inflammation and local immune response^15^. In addition, HNSCC might have more shared common antigens related to the skin. The higher percentage of patients with HNSCC recruited in the current study than those reported in the literature might be due to the ethnic and geographic differences in patient population. HNSCC is a major cancer in Taiwanese male population^14^.

The interference of concomitant treatments, especially molecular targeted therapy, is delineated and is significantly correlated with a higher incidence of cutaneous irAEs. The interference or interaction between concomitant medication and immunotherapy has been demonstrated in the background of literature regarding cutaneous irAEs^5,13^, but is still often overlooked. The detailed mechanism for such phenomenon has not been well elucidated. The molecular targeted therapy may impair epidermal differentiation and cause tissue inflammation. These might be the possible explanations for the increased risk of cutaneous irAEs in patients receiving concomitant molecular targeted therapy and immunotherapy. Furthermore, given the fact that targeted therapy itself is associated with frequent dermatologic toxicities^6,15^, as well as that the emerging regimens in combination with immunotherapy are commonly carried out in current clinical scenario, the effect of concomitant medication should be taken seriously, especially in further related studies analyzing adverse events.

Detailed subtype analysis in our study revealed that MPE, pruritus, and eczematous eruption were the most prevalent cutaneous irAEs regardless of numbers of ICIs used. Similar results can be found in one of the largest meta-analyses of 125 clinical trials, in which the incidence of pruritus and rash were 10.6 and 9.3%, respectively^16^. Less common cutaneous irAEs, including psoriasiform eruption, lichenoid eruption, and BP, were also noted, in the forms of exacerbation or occurrence of psoriatic skin lesions or blisters, which could appear rapidly or only after several months of treatment^8^, but lacked actual incidence due to the few case numbers^17,18^. It has been reported that the development of lichenoid eruptions and BP were more commonly associated with anti-PD-1/PD-L1 antibody therapy and not with anti-CTLA-4 antibody therapy^10,11,19,20^. In the current study, we observed 2 lichenoid eruptions and 1 BP only in the pembrolizumab group with the incidences of 0.75% and 0.38% respectively, which were similar to the previous review (0.81% for lichenoid eruptions and 0.29% for pemphigoid)^12^.

The histologic examination may aid in diagnosing lichenoid cutaneous irAE, and the incidence was likely underestimated since skin biopsy was seldom carried out in mild clinical presentation, which mimics MPE or eczematous eruption in appearance, and the incidence reported in one single institute cohort was 17.1%^8,20,21^. However, in cases with a biopsy performed, lichenoid dermatitis (50%) was the most common histologic pattern followed by spongiotic dermatitis (40%)^22^. In the current study, only 13 of the 128 patients had received skin biopsies (10.2%), which might lead to misclassification and underestimation of lichenoid cutaneous irAEs.

The occurrence of some cutaneous irAEs may be correlated with a better therapeutic response, in terms of objective response (vitiligo), progression-free survival (BP, ircAEs all together), or overall survival (BP, MPE, and vitiligo) in different subsets of clinical trials^8,23^. A recent report also showed development of ≥ 1 of the 3 cutaneous irAEs (eczema, lichenoid reaction, or vitiligo-like depigmentation) is associated with improved progression-free survival in melanoma patients treated with pembrolizumab or nivolumab^24^. Survival advantage in terms of overall survival was demonstrated in our patients with cutaneous irAEs, compared to those without, and further subgroup analysis showed that any subgroup of patients with cutaneous irAEs had a longer survival time compared to those without, while no superiority of any type was established. Vitiligo, which is regarded as a possibly positive correlation with the outcome, mainly presents itself only in melanoma patients at approximately 7.5–11% in nivolumab- and pembrolizumab-treated patients, and was seen as lower in those who were treated with ipilimumab^8^. Although it was exceptional in other forms of cancer, reporting 3.26% in a pooled meta-analysis of various cancers^16^, the presence of vitiligo when treating solid cancers like lung adenocarcinoma was reported with partial tumor resolution^7,25^. Only one melanoma patient in our study developed vitiligo at three months after the administration of nivolumab, with the sustained response of a stable disease. The presence of vitiligo persisted for more than 18 months even after discontinuing nivolumab.

Severe cutaneous irAEs including SJS/TEN and drug reaction with eosinophilia and systemic symptoms induced by immunotherapy have been delineated in a few case reports and have required treatment cessation^5,26–29^. Two cases of pembrolizumab-related SJS were documented in the current study with a latency of 28 and 42 days after pembrolizumab, while high-risk medications like antibiotics were concomitantly used during the treatment period. Recently, we also reported another patient suffering from SJS after 18-cycles of nivolumab with concomitantly used esomeprazole^30^. The culprit medication was later confirmed to be esomeprazole.

Our study had some limitations. First, this study was a single center retrospective study, and the median follow-up duration was relatively short. However, we have tried to do our best to recruit all the eligible patients into the analysis. The relatively short duration of median follow-up time was resulted partly from the mortality of these advanced cancer patients. Second, patients with mild presentations may not have a dermatology consultation thus leading to a referral bias and a possible underestimated incidence rate of cutaneous irAEs.

In conclusion, MPE, pruritus, and eczematous eruption are the most common forms of cutaneous irAE in our study. Concomitant treatments, especially molecular targeted therapies, may increase the incidence of cutaneous irAEs. Having any type of cutaneous irAE is correlated with a longer overall survival.

## Methods

### Patients and diagnosis of cutaneous irAEs

Patients receiving ICIs including anti-PD-1 (nivolumab and pembrolizumab), anti-CTLA-4 (ipilimumab), and anti-PD-L1 (atezolizumab) at the National Taiwan University Hospital between January 1, 2014, and December 31, 2017, were retrospectively identified from the Integrated Medical Database of National Taiwan University Hospital. This study has been approved by the Research Ethics Committee of the hospital (201705019RINB) and was performed in accordance with the guidelines and regulations of the Ethics Committee. Patients were excluded if only one course of ICI was administered, or they were followed for fewer than three weeks. The informed consent was obtained from all the participants. Detailed medical records were reviewed until June 30, 2018, via electronic medical record system, collecting patient demographics including cancer diagnosis, concurrent medication used, and diagnoses of cutaneous irAEs made by the dermatologists in both out-patient departments and in-patient consultation. Symptoms onset or aggravated after administration of ICIs in medical records during the study period were collected. The final diagnoses of the cutaneous irAEs were based on the clinical evaluation which were judged by and were agreed among the participating dermatologists. The histopathology findings were served as supporting information in some cases if a skin biopsy was performed. Finally, the status of survival and date of death were retrieved from the database of the Ministry of Health and Welfare of Taiwan, dating until June 30, 2018.

### Statistical analysis

Baseline demographics and different types of cutaneous irAEs were summarized with descriptive statistics, demonstrating the absolute case numbers, relative frequencies, mean, and standard deviation. The chi-squared test was used to compare categorical data. Fisher’s exact test was applied when at least one expected frequency was less than 5. A two-sided P value of < 0.05 was considered statistically significant. The multivariable logistic regression analysis was used when comparing the effects of different factors on the development of cutaneous irAEs. The Kaplan–Meier plot and log-rank test were used to demonstrate the relationship between survival and adverse events, and the Bonferroni correction was applied for subgroup analysis adjustment. A conditional landmark analysis was applied to minimize the guarantee-time bias. A Cox regression model was used to examine the potential confounders such as combination/sequential therapy, age, gender, and cancer types. Patients with cutaneous irAEs, the time periods before the development of cutaneous irAEs were subtracted from the total survival time. These subtracted values were then compared with those of patients without cutaneous irAEs. To Statistical analyses were performed using SAS software (version 9.4 [TS1M1] https://support.sas.com/software/94).

## Ethical approval

This study has been approved by the Research Ethics Committee of National Taiwan University Hospital (201705019RINB).

## Supplementary Information

Supplementary Information 1.

Supplementary Information 2.

Supplementary Information 3.

## Data Availability

The datasets generated during and/or analysed during the current study are available from the corresponding author on reasonable request.

## Abbreviations

Anti-CTLA-4: Anti-cytotoxic T-cell lymphocyte-associated antigen-4
Anti-PD-1: Anti-programmed death-1
Anti-PD-L1: Anti-programmed death ligand-1
BP: Bullous pemphigoid
HCC: Hepatocellular carcinoma
HNSCC: Head and neck squamous cell carcinoma
ICI: Immune checkpoint inhibitor
irAE: Immune-related adverse event
MPE: Maculopapular eruption
NSCLC: Non-small cell lung cancer
SJS/TEN: Stevens-Johnson syndrome/toxic epidermal necrolysis
