# Supplementary figures and images for: Cutaneous immune-related adverse events among Taiwanese cancer patients receiving immune checkpoint inhibitors link to a survival benefit
Source: Sci Rep. 2022 Apr 29;12:7021. doi: 10.1038/s41598-022-11128-5 (PMC9055047; doi:10.1038/s41598-022-11128-5)

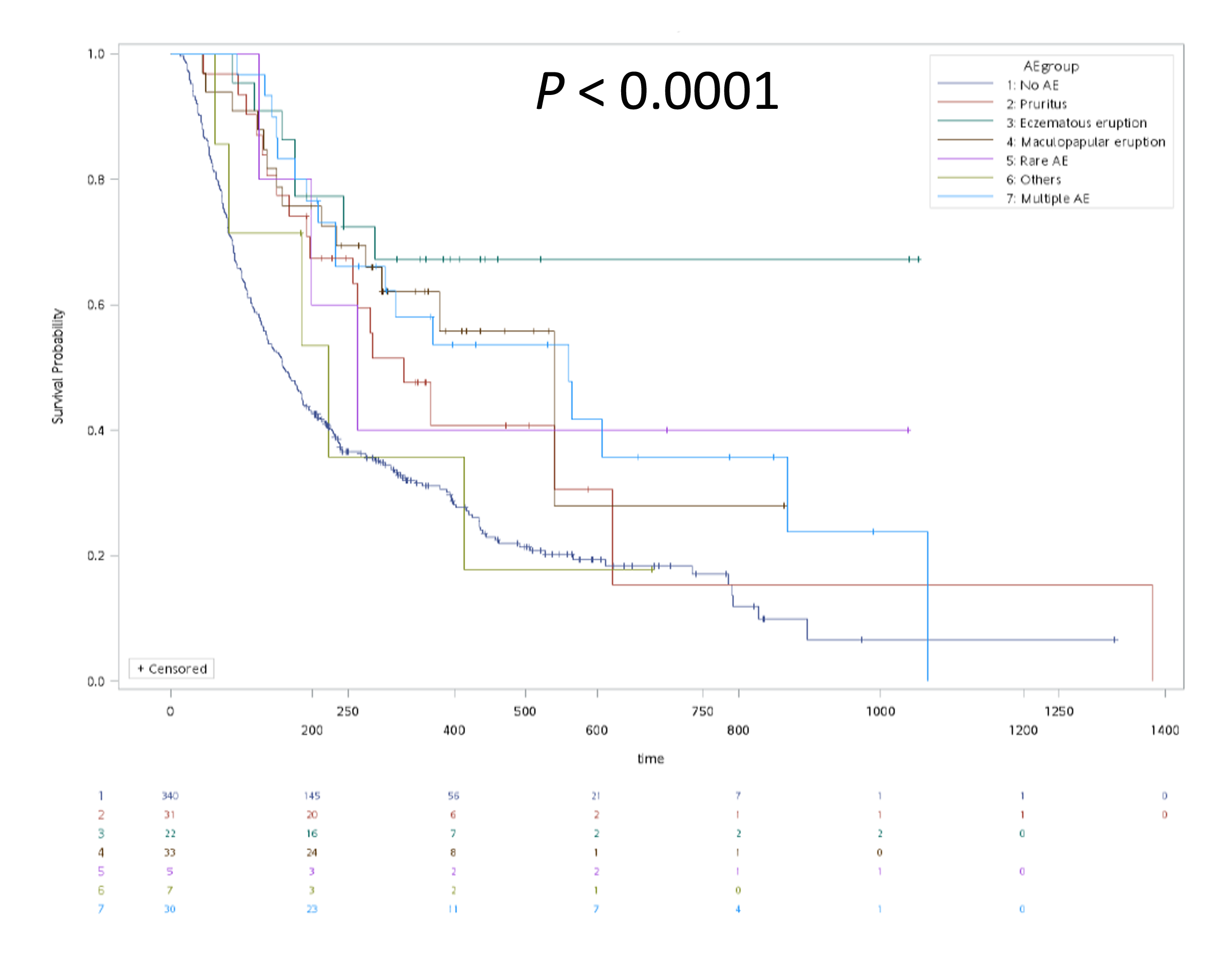

Supplement: Supplementary file 1 — Supplementary Information 1. [file 41598_2022_11128_MOESM1_ESM.tif]

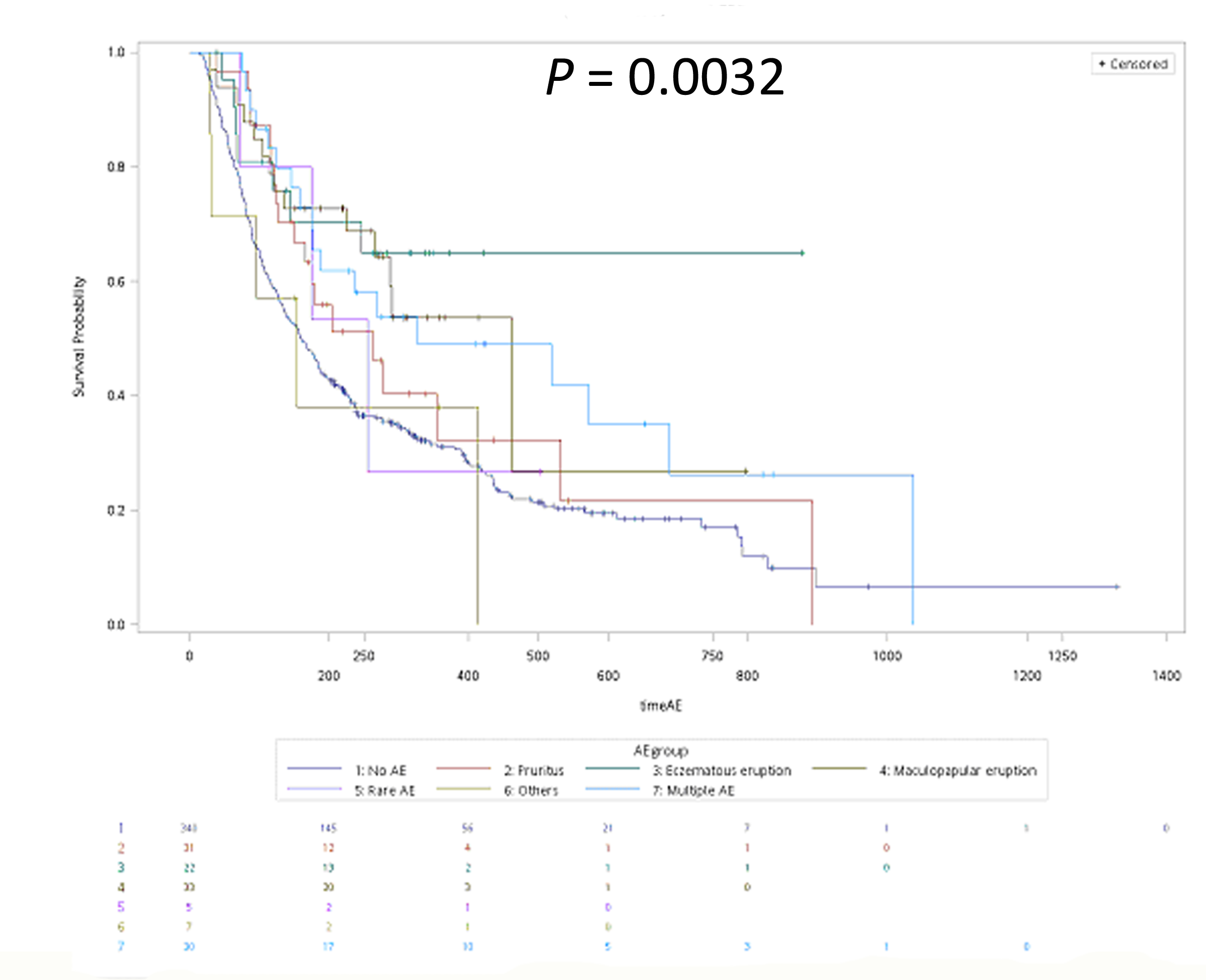

Supplement: Supplementary file 2 — Supplementary Information 2. [file 41598_2022_11128_MOESM2_ESM.tif]
