# Supplementary material for: Cutaneous immune-related adverse events among Taiwanese cancer patients receiving immune checkpoint inhibitors link to a survival benefit
Source: Sci Rep. 2022 Apr 29;12:7021. doi: 10.1038/s41598-022-11128-5 (PMC9055047; doi:10.1038/s41598-022-11128-5)
Supplement: Supplementary file 3 — Supplementary Information 3. [file 41598_2022_11128_MOESM3_ESM.doc]

**Cutaneous immune-related adverse events among Taiwanese cancer patients receiving immune checkpoint inhibitors link to a survival benefit**

Yung-Tsu Cho, M.D.; Yi-Tsz Lin, M.D.; Che-Wen Yang, M.D. M.S.; Chia-Yu Chu, M.D. Ph.D.

**Supplementary Figure Legends**

**Supplementary Figure 1**. Subgroup analysis showed that the survival time of patients without cutaneous irAEs was shorter than any subgroup of patients with different cutaneous irAEs, while no superiority of any type was established after application of the Bonferroni correction.

**Supplementary Figure 2**. Subgroup analysis of the data after applying the conditional landmark analysis showed that the survival time of patients without cutaneous irAEs was shorter than any subgroup of patients with different cutaneous irAEs, while no superiority of any type was established after application of the Bonferroni correction.
